# Supplementary material for: Biological Evaluation of 3-Benzylidenechromanones and Their Spiropyrazolines-Based Analogues
Source: Molecules. 2020 Apr 1;25(7):1613. doi: 10.3390/molecules25071613 (PMC7180617; doi:10.3390/molecules25071613)
Supplement: Supplementary file 1 [file molecules-25-01613-s001.pdf]

Supporting information

Biological evaluation of 3-benzylidenechromanone and their spiropyrazolines-based analogues

Angelika A. Adamus-Grabicka[1], Magdalena Markowicz-Piasecka[2], Marcin Cieślak[3], Karolina Królewska-Golińska[3], Paweł Hikisz[4], Joachim Kusz[5], Magdalena Małecka[6] and ElzbietaBudzisz[1,*]

[1] Department of Cosmetic Raw Materials Chemistry, Faculty of Pharmacy, Medical University of Lodz, Muszynskiego 1, 90-151 Lodz, Poland; angelika.adamus@umed.lodz.pl

[2] Laboratory of Bioanalysis, Department of Pharmaceutical Chemistry, Drug Analysis and Radiopharmacy, Medical University of Lodz, Muszynskiego 1, 90-151 Lodz, Poland; magdalena.markowicz@umed.lodz.pl

[3] Centre of Molecular and Macromolecular Studies, Polish Academy of Sciences, Sienkiewicza 112, 90-363 Lodz, Poland; marcin@cbmm.lodz.pl; kkrolews@cbmm.lodz.pl

[4] Department of Molecular Biophysics, Faculty of Biology and Environmental Protection, University of Lodz, Pomorska 141/143, 90-236, Lodz, Poland; pawel.hikisz@biol.uni.lodz.pl

[5] Institute of Physics, University of Silesia, Uniwersytecka 4, 40-007 Katowice, Poland; joachim.kusz@us.edu.pl

[6] Department of Physical Chemistry, Theoretical and Structural Chemistry Group, Faculty of Chemistry, University of Lodz, Pomorska 163/165, 90-236 Lodz, Poland; magdalena.malecka@chemia.uni.lodz.pl

* Correspondence: elzbieta.budzisz@umed.lodz.pl; Tel.: +42-272-55-95

S1. Chemistry

IR spectrum of compounds show bands at 1697, 1684, 1677, 1665, 1663 cm-1 for ν (C=O) group. The bands typical for aromatic rings were observed at 1604, 1578, 1568 1506 cm-1. The N=N stretch frequency were detected at 1472 cm^{-1} for compound **2** and **10**, at 1460 cm^{-1} for compound **6**, at 1468 cm^{-1} for **4** and at 1464 cm^{-1} for **8**. The C-N group vibration was observed at 1303 cm-1for compound **6** and 1311 cm^{-1} for **8**. For **2**, **10** and **8** structures the IR spectrum presented ν (C-N) bands at 1307 and 1309 cm^{-1}. The ^{1}H NMR spectrum for compound **1** revealed the presence of OCH_3 protons at δ 2.3 ppm. The signals around δ 6.9 – 7.8 ppm corresponding to aromatic protons and those at δ 6.3 ppm to the C2-H proton. At δ 7.7 ppm the signal for =CH as singlet was observed. The mass spectrum confirmed the structure of the **1** by the presence of $[M+H]^+$ at m/z = 343. For compound **2** the ^{1}H NMR spectrum showed OCH_3 protons at 3.7 ppm, with signals around δ 6.9 – 7.7 ppm corresponding to aromatic protons. The proton at C2 was detected at δ 5.9 ppm as a singlet. Signal at δ 4.2 is typical of CH_2. The expected spiropyrazoline structure **2** was confirmed for the product by the mass spectrometry $[M+H]^+$ at m/z = 385. In the spectrum of benzylideneflavanone **5** there is a signal of proton attached to C2 carbon atom at δ 6.3 ppm. A singlet for =CH was observed at δ 7.7 ppm. The signals around δ 6.9 – 7.8 ppm corresponding to the aromatic protons. The mass spectrum confirmed the structure of the **5** by the presence of the band of the $[M+H]^+$ at m/z =313. In the spectrum of spiropyrazoline **6** there are a characteristic proton signals at δ 4.2 ppm and δ 4.2 ppm from CH_2. The signals around δ 7.1 – 7.7 ppm corresponding to aromatic protons. The proton at C2 was detected at δ 5.9 ppm as a singlet. The mass spectrum confirmed the structure of the **6** by the presence of $[M+H]^+$ at m/z = 355. The spectrum of compound **9** is similar to **1.** At δ 6.2 ppm the signal for C2-H as singlet was observed for **9**. The signals around δ 6.9 – 7.8 ppm corresponding to aromatic protons and the signal δ 8.3 ppm for =CH was observed. The mass spectrum confirmed the structure of the **9** by the presence of $[M+H]^+$ at m/z = 342. The structure of compound **10** was confirmed by the signals in the proton spectrum. The protons of methoxy group were observed at δ 3.7 ppm. At δ 5.5 ppm a singlet for the C2-H group was observed. The signals present around δ 6.8 – 7.8 ppm corresponding to aromatic protons. The spiropyrazoline structure **10** was confirmed by the product of the mass spectrometry analysis $[M+H]^+$ at m/z = 384. In the ^{13}C NMR spectrum the characteristic methoxy group signal was observed around δ 55.2 – 58.4 for compounds **1**, **2** and **9**. The signals from C2-H group were observed around δ 77.3 – 79.5 ppm for **1**, **2**

and **5**, **6** while the signal from C2-H in **9** and **10** was observed at 77.7 and 81.5 ppm. In addition the ^{13}C NMR spectrum showed the presence of signals from CH_{arom} and C_{arom} around 112.4 – 159.6 ppm and C=O signal around 185 ppm which indicates the structure of the compounds: benzylideneflavanones and spiroflavanones. For compounds **3** and **4** the ^{13}C NMR spectrum showed signal at δ 55.5 and 55.3 ppm attributed to a OCH_3 group. The signal at 128.9 ppm for =CH was observed. The spectrum showed the presence of signals at 77.0 and 77.3 ppm attributed to CH_2 group and signals located at 182.3 and 186.2 ppm attributed to a C=O group. The signals about 98.0 – 161.7 ppm from CH_{arom} and C_{arom} were also found. For the compounds **7** the ^{13}C NMR spectrum showed the signal at 77.0 ppm from CH_2 group and the signal located at 134.5 ppm attributed to =CH group. The spectrum of **7** showed the presence of CH_{arom} and C_{arom} signals about 108.2 and 155.9 ppm. The signal for C=O at 182.3 ppm was observed too. In the spectrum of **8** the signals from CH_{arom}, C_{arom} and C=O were also found. The signals located at 77.3 ppm and 85.7 ppm attributed to two CH_2 groups and a CH signal at 42.4 ppm. In the spectrum of **3** singal at δ 3.8 (singlet) comes from methoxy group protons. At δ 4.9 ppm a singlet for the =C-H was observed. At δ 7.0 – 7.5 ppm the signals for aromatic protons were observed. A doublet for C2-H protons was observed at δ 7.6 ppm. The mass spectrum confirmed the structure of the **3** by the presence of $[M+H]^+$ at m/z = 267. The structure of compound **4** was confirmed by typical signals in the ^{1}H NMR spectrum. At δ 4.58 ppm a doublet for the C2-H protons was observed. The characteristic signals δ 6.9 – 7.7 ppm correspond to the aromatic protons. At δ 3.8 ppm a singlet for methoxy group protons was noted. The characteristic proton signals were observed at δ 5.1 ppm and δ 5.2 ppm from CH_2. The spiropyrazoline **4** structure was confirmed by the mass spectrometry ($[M+H]^+$ at m/z = 309).The ^{1}H NMR spectrum for compound **7** revealed the presence of a singlet from the =C-H proton at δ 5.4 ppm. A doublet for C2-H was observed at δ 7.6 ppm. The signals from aromatic protons were observed at δ 7.0 – 7.5 ppm. For compound **8** the ^{1}H NMR spectrum showed the signals around δ 7.1 – 7.7 ppm corresponding to the aromatic protons. Signals at δ 4.9 and δ 5.1 ppm was detected as doublet from C2 carbon atom. At δ 4.1 ppm and δ 4.2 ppm the signals for CH_2 were noted. The mass spectrum confirmed the structure of the **7** by the presence of the band of the $[M+H]^+$ at m/z = 237. In the mass spectrum a signal at m/z = 279.4 was present which confirmed the structure of **8** ($[M+H]^+$).

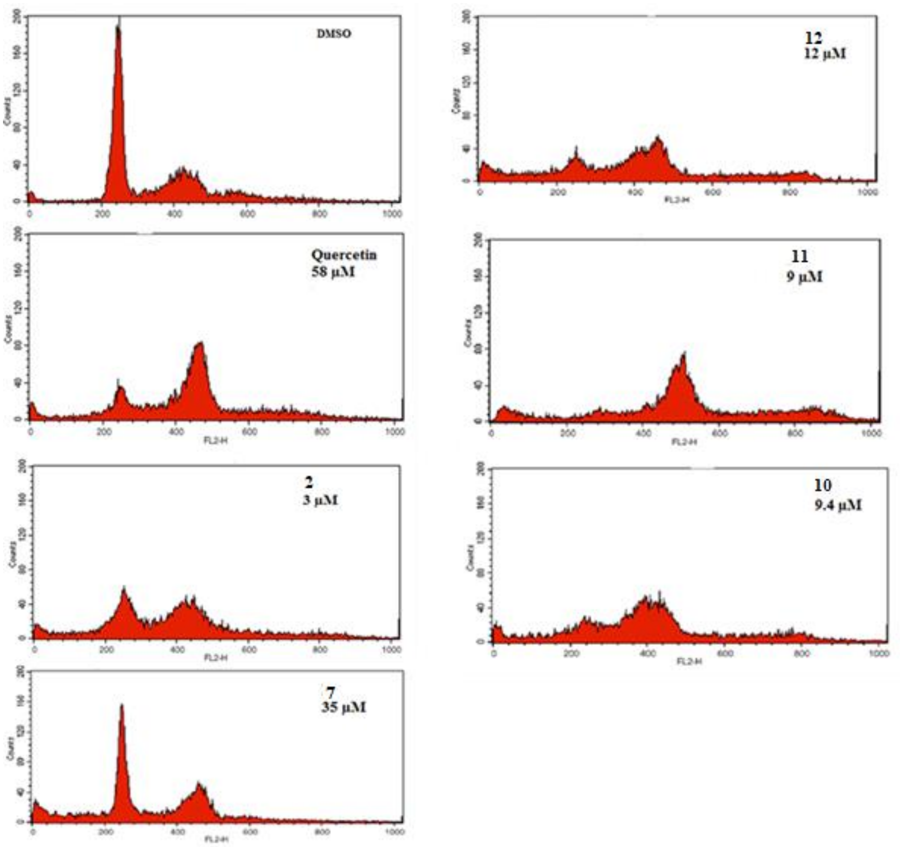

Figure S2. The effect of the test compounds and quercetin on the cell cycle in HL-60 cells. Histograms of DNA content in HL-60 cells treated with compounds at a concentration of 1xIC$_{50}$.

	**HL 60**	**DMSO control**	**Quercetin (58)**	**Quercetin (29)**	**1 (3)**	**1 (1.5)**	**7 (35)**	**7 (18)**	**10 (9.4)**	**10 (4.7)**	**11 (9)**	**11 (4.5)**	**12 (12)**	**12 (6)**
**G1**	45,20%	71,82%	12,78%	15,93%	13,57%	38,88%	51,06%	62,77%	25,68%	59,30%	5,16%	8,45%	28,24%	21,08%
		68,77%	10,40%	12,59%	29,04%	8,15%	49,68%	52,64%	6,79%	21,37%	1,17%	3,45%	10,82%	17,44%
**S**	39,24%	21,68%	41,15%	23,57%	17,63%	21,38%	22,89%	18,79%	32,45%	20,39%	26,86%	27,42%	27,06%	22,13%
		19,26%	37,60%	26,40%	32,40%	24,69%	24,20%	24,16%	34,06%	40,07%	31,30%	42,23%	29,00%	34,60%
**G2/M**	15,56%	6,50%	46,07%	60,50%	68,86%	39,74%	26,04%	18,44%	41,88%	20,31%	67,98%	64,13%	44,70%	56,79%
		11,97%	52,00%	61,01%	38,56%	67,16%	26,13%	23,20%	59,14%	38,56%	67,53%	54,32%	60,18%	47,96%
**G1**	45,20%	70,30%	11,59%	14,26%	21,31%	23,52%	50,37%	57,71%	16,24%	40,34%	3,17%	5,95%	19,53%	19,26%
**S**	39,24%	20,47%	39,38%	24,99%	25,02%	23,04%	23,55%	21,48%	33,26%	30,23%	29,08%	34,83%	28,03%	28,37%
**G2/M**	15,56%	9,24%	49,04%	60,76%	53,71%	53,45%	26,09%	20,82%	50,51%	29,44%	67,76%	59,23%	52,44%	52,38%

Figure S3. The cell cycle of the tested compounds; number of repetition n=2.

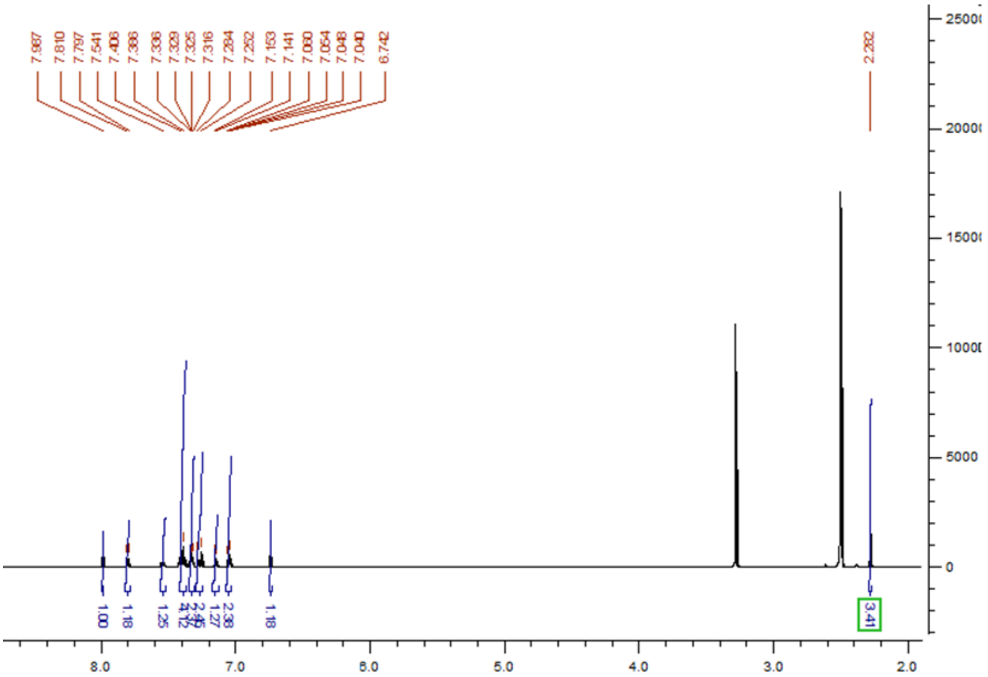

Figure S4. [1] ***H* NMR spectrum of compound 1** (DMSO-d_6 2.50 ppm; HDO 3.25 ppm)

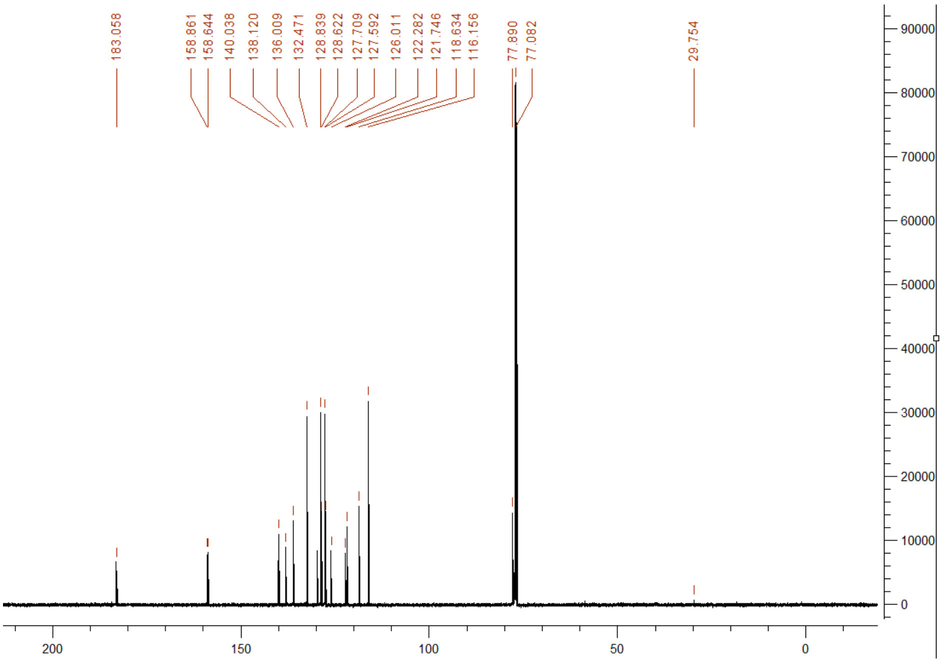

Figure S4a. ^{13}***C* NMR spectrum of compound 1** ($CDCl_3$ 77.0 ppm)

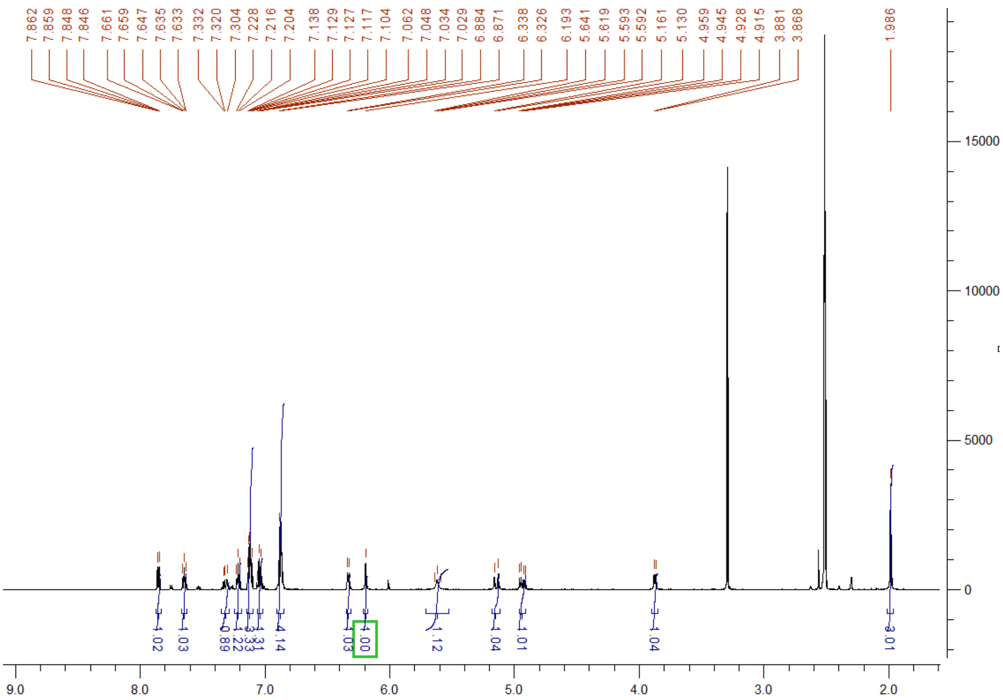

Figure S5. **^{1}H NMR spectrum of compound 2** (DMSO-d_6 2.50 ppm; HDO 3.25 ppm)

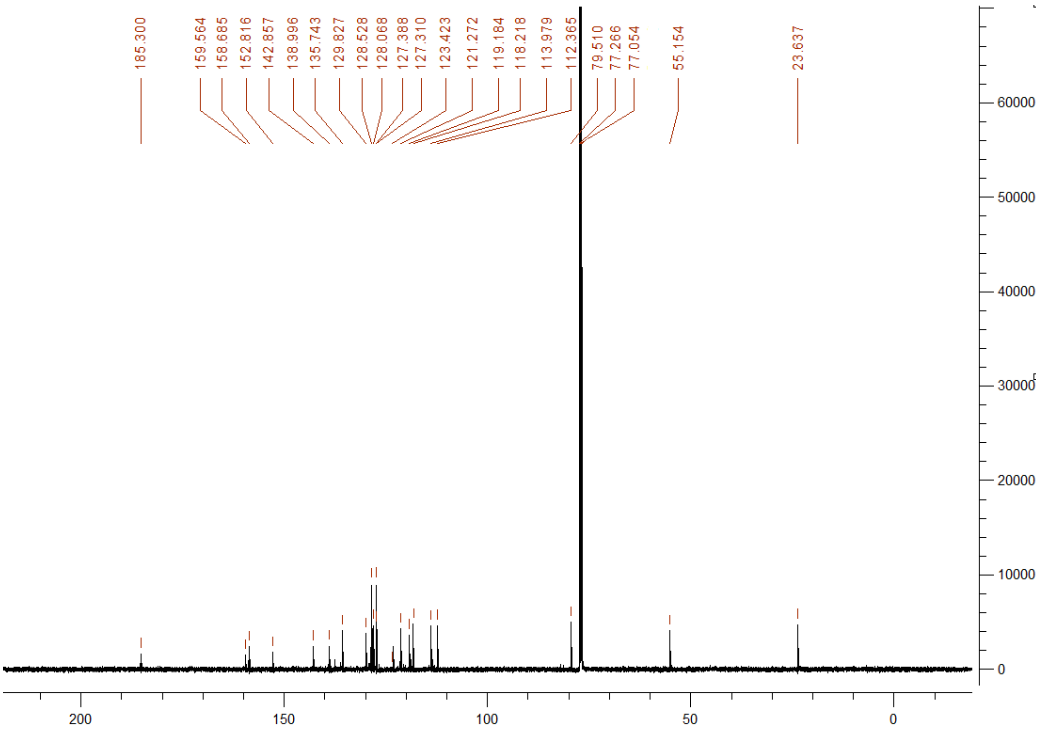

Figure S5a. **^{13}C NMR spectrum of compound 2** ($CDCl_3$ 77.0 ppm)

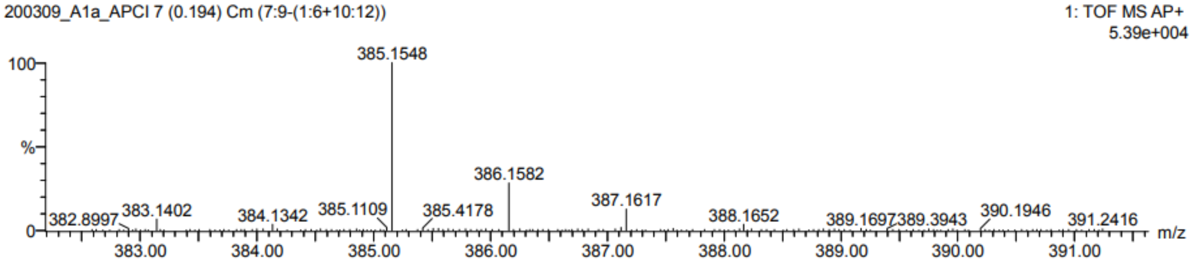

Figure S5b. HRMS spectrum of compound 2.

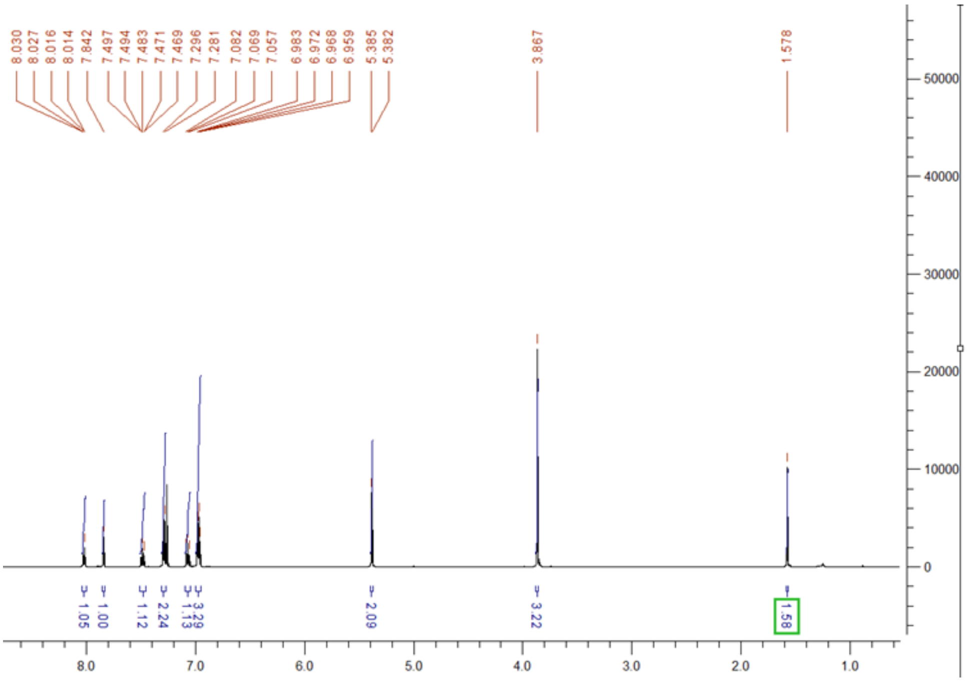

Figure S6. **[1]*H* NMR spectrum of compound 3** (CDCl3 7.26 ppm)

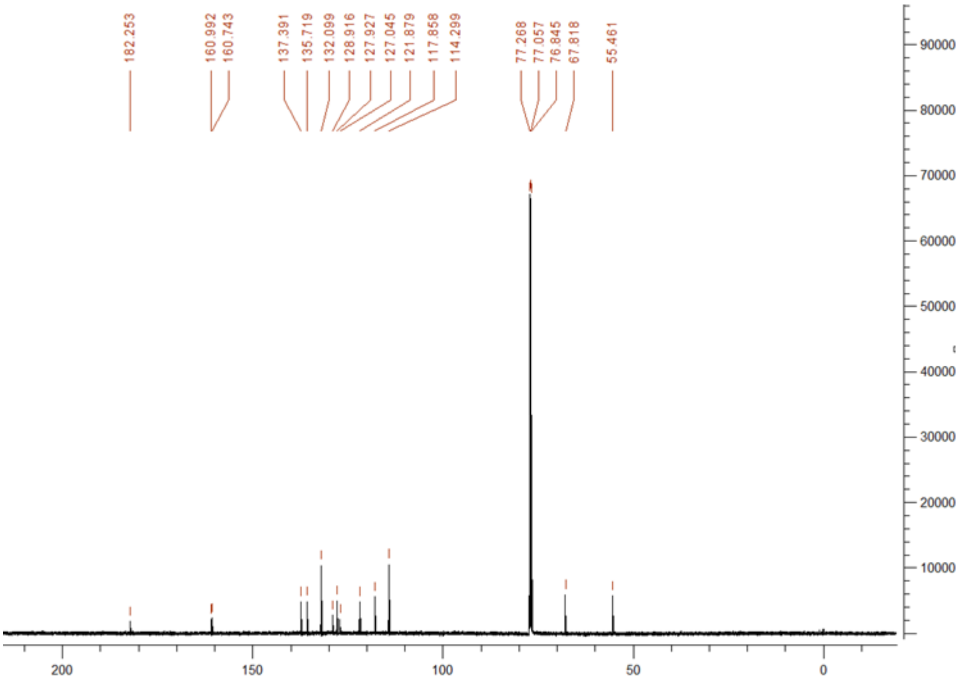

Figure S6a. ^{13}C **NMR spectrum of compound 3** (CDCl3 77.0 ppm)

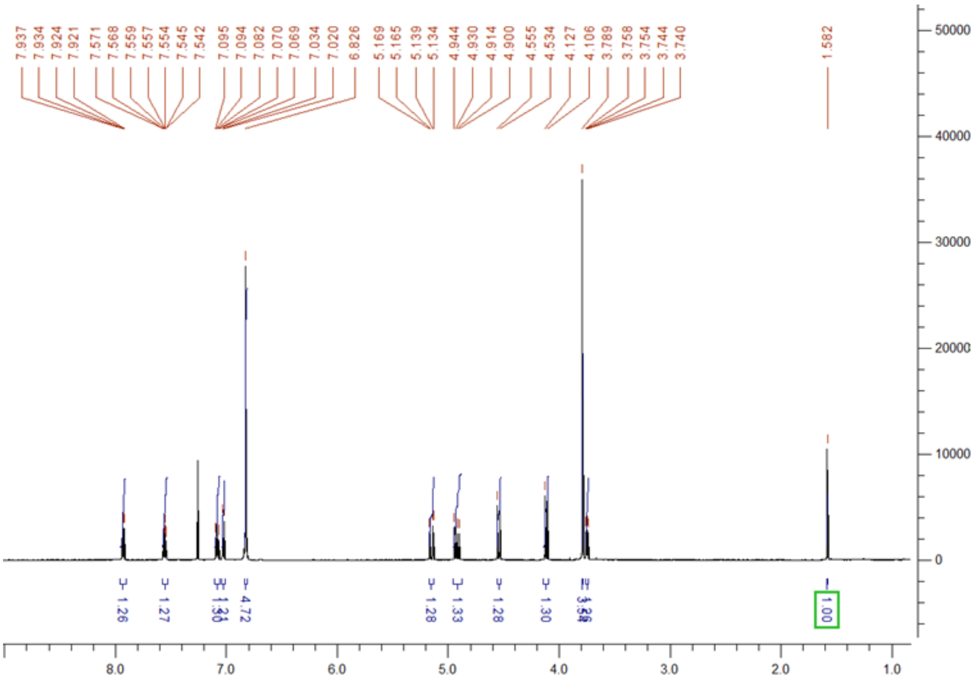

Figure S7. ^{1}H **NMR spectrum of compound 4** (CDCl3 7.26 ppm)

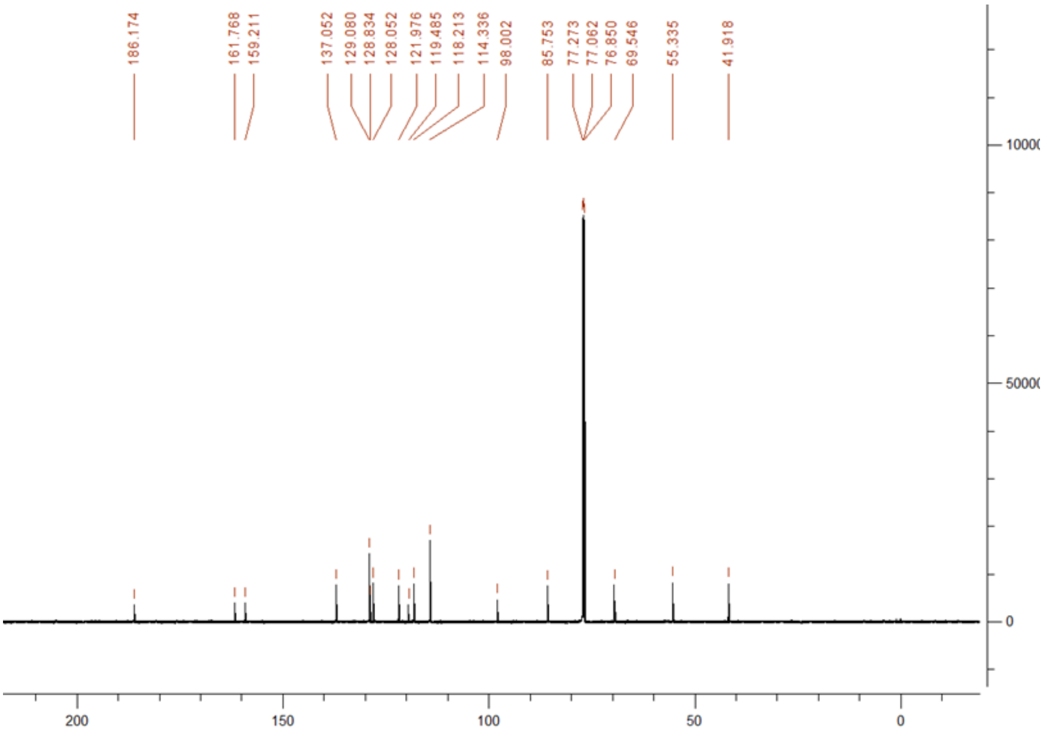

Figure S7a. ^{13}C **NMR spectrum of compound 4** (CDCl3 77.0 ppm)

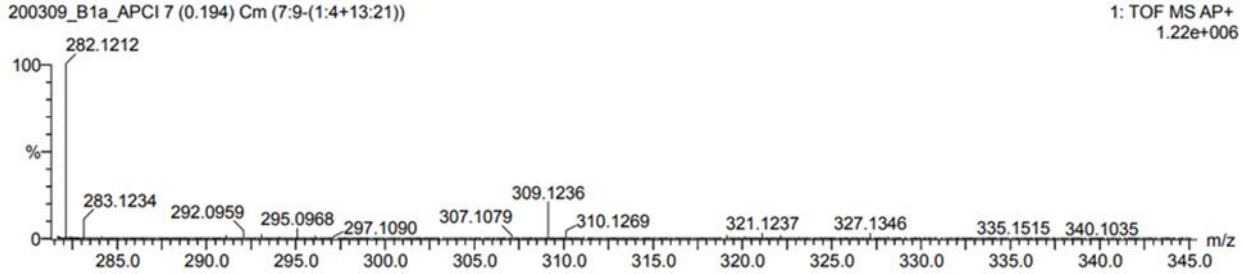

Figure S7b. HRMS spectrum of compound 4.

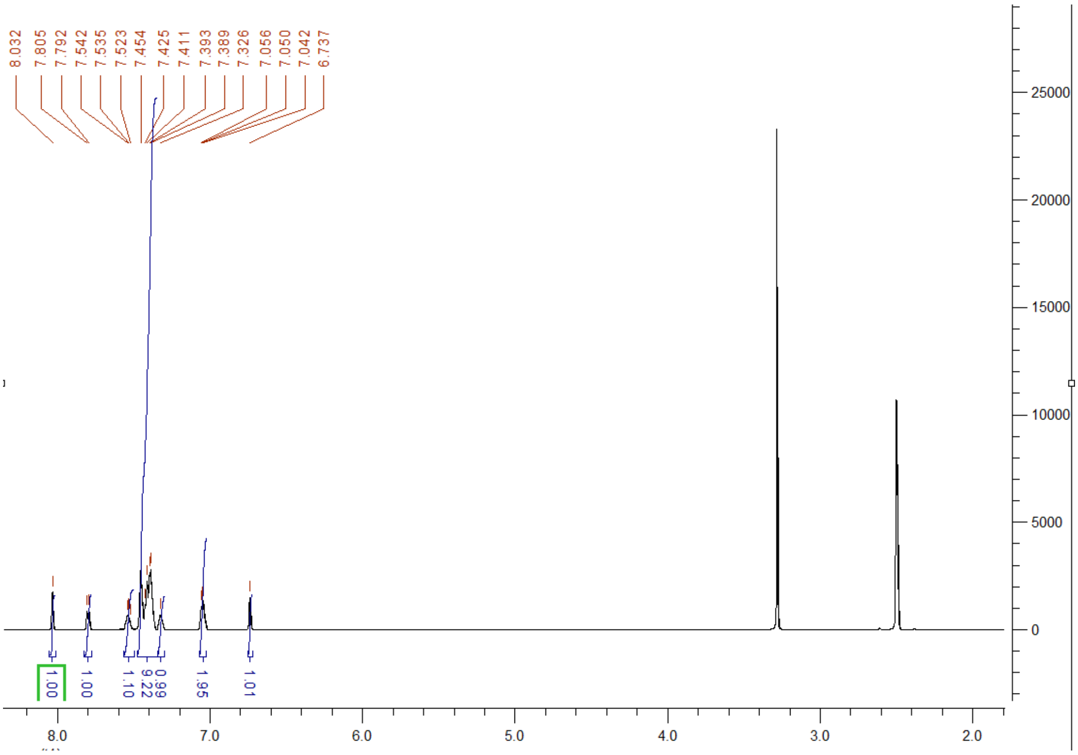

Figure S8. ^{1}*H* NMR spectrum of compound 5 (DMSO-d$_6$ 2.50 ppm; HDO 3.25 ppm)

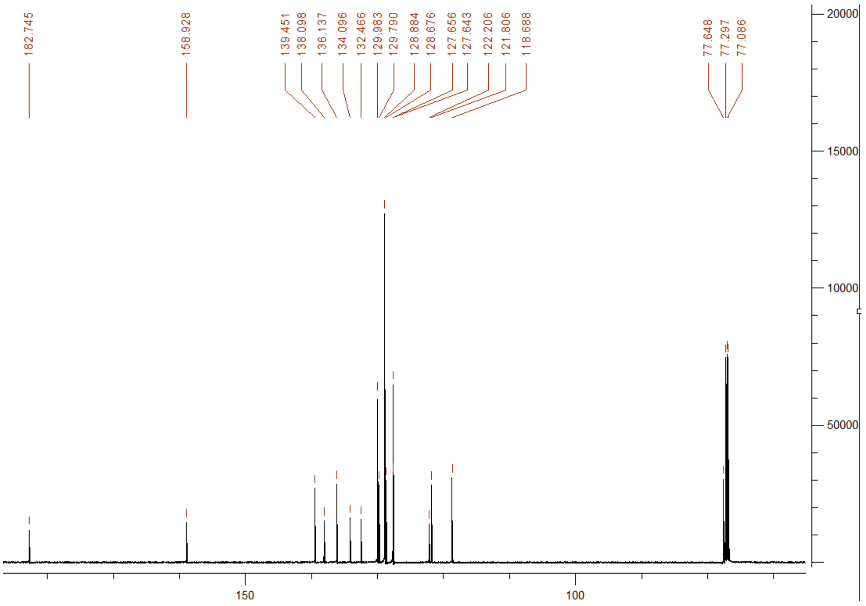

Figure S8a. ^{13}C **NMR spectrum of compound 5** (CDCl3 77.0 ppm)

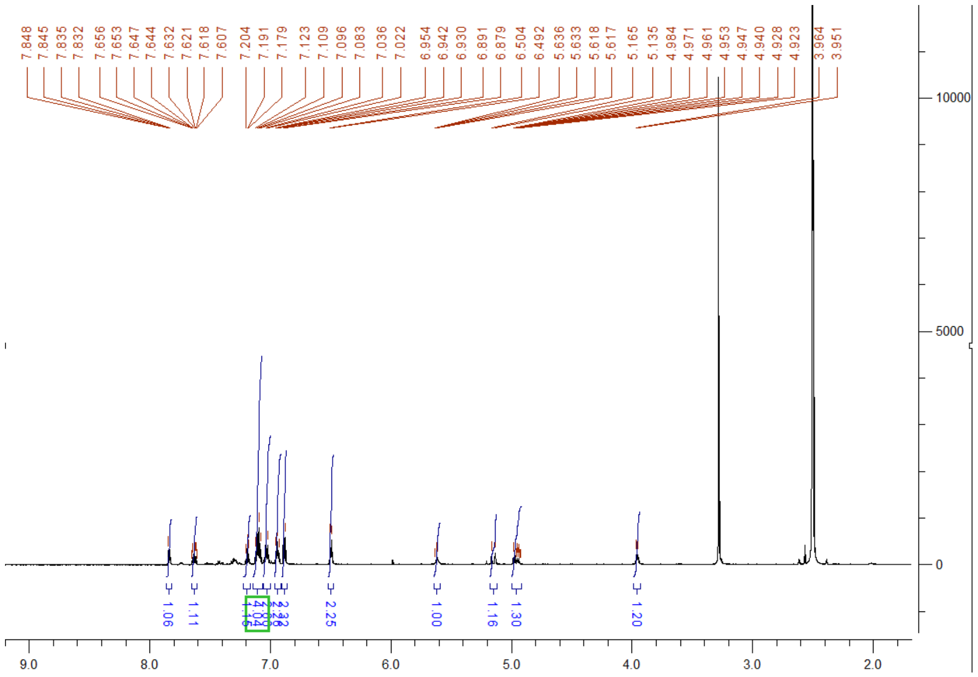

Figure S9. ^{1}H **NMR spectrum of compound 6** (DMSO-d6 2.50 ppm; HDO 3.25 ppm)

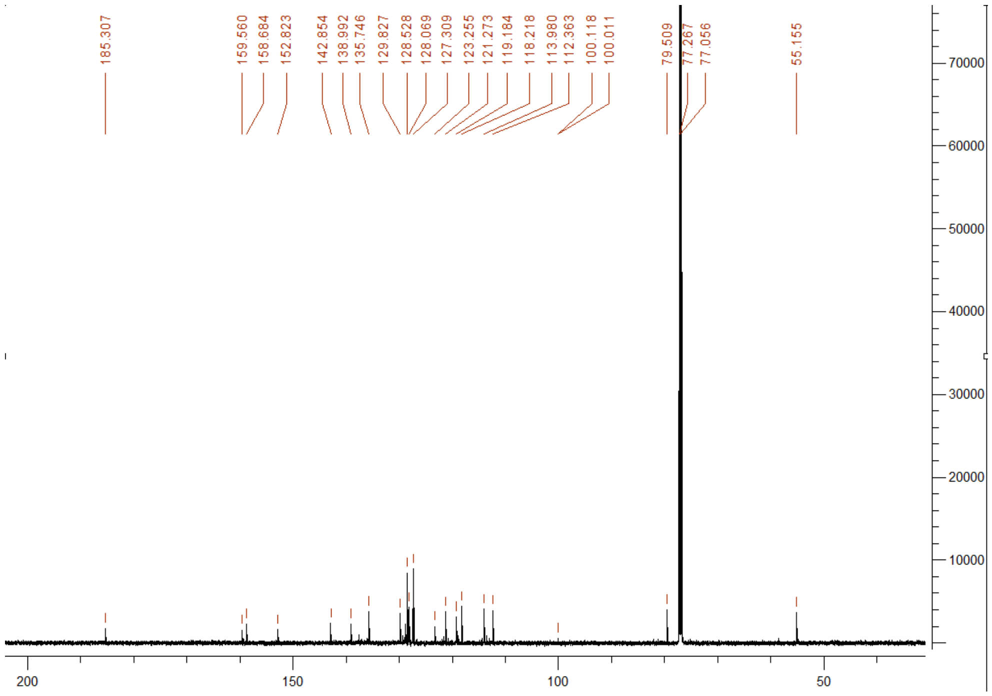

Figure S9a. ^{13}C **NMR spectrum of compound 6** (CDCl3 77.0 ppm)

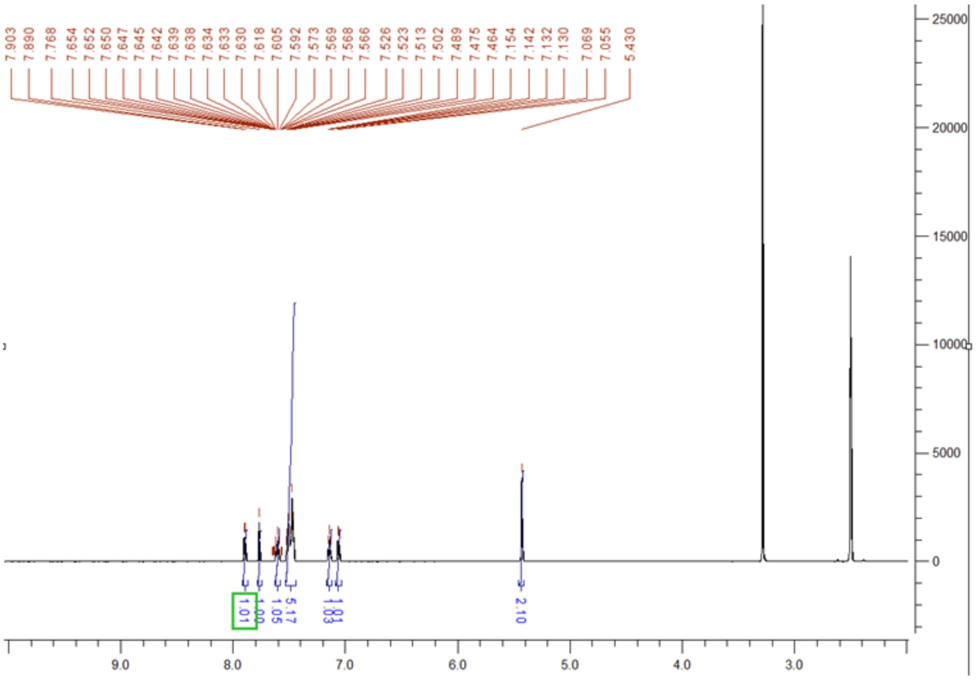

Figure S10. ^{1}***H* NMR spectrum of compound 7** (DMSO-d6 2.50 ppm; HDO 3.25 ppm

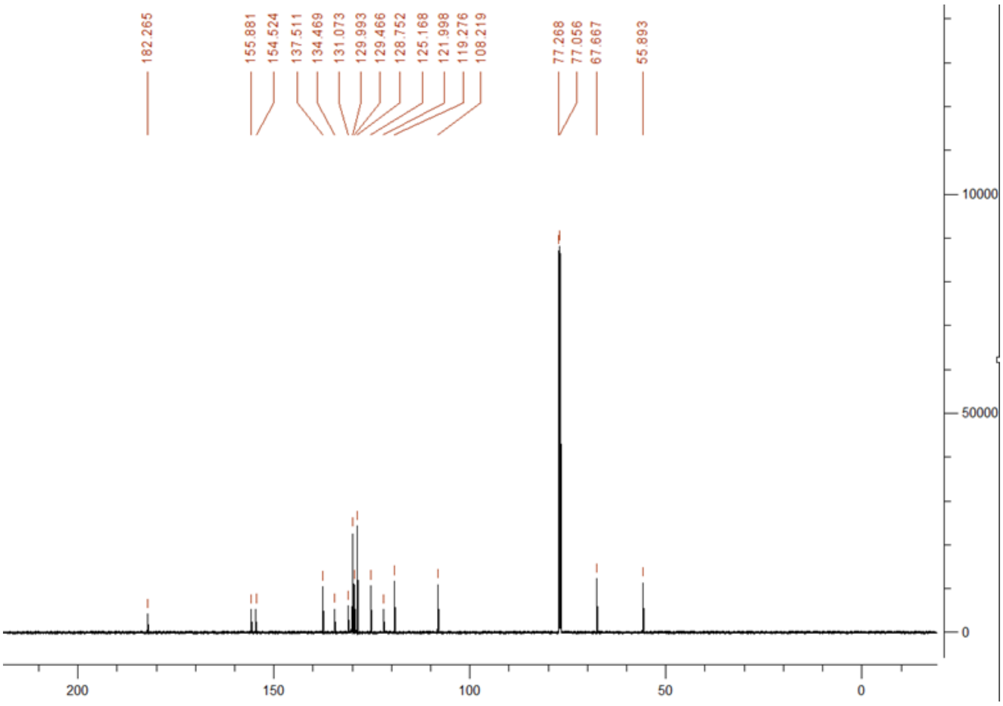

Figure S10a. ^{13}***C* NMR spectrum of compound 7** ($CDCl_3$ 77.0 ppm)

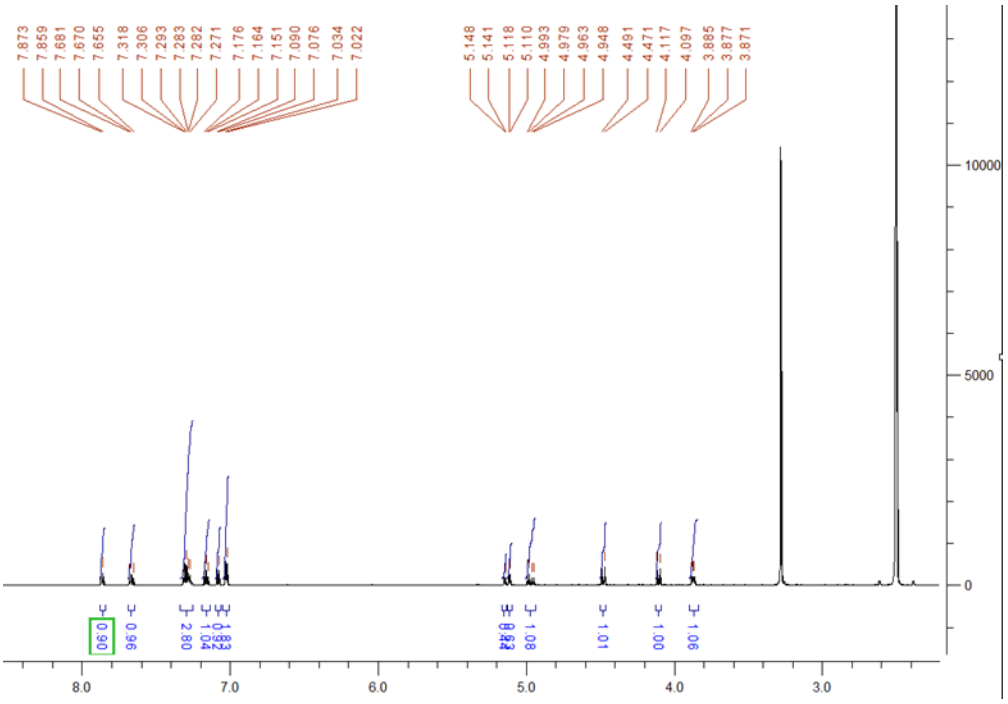

Figure S11. ^{1}H **NMR spectrum of compound 8** (DMSO-d$_6$ 2.50 ppm; HDO 3.25 ppm)

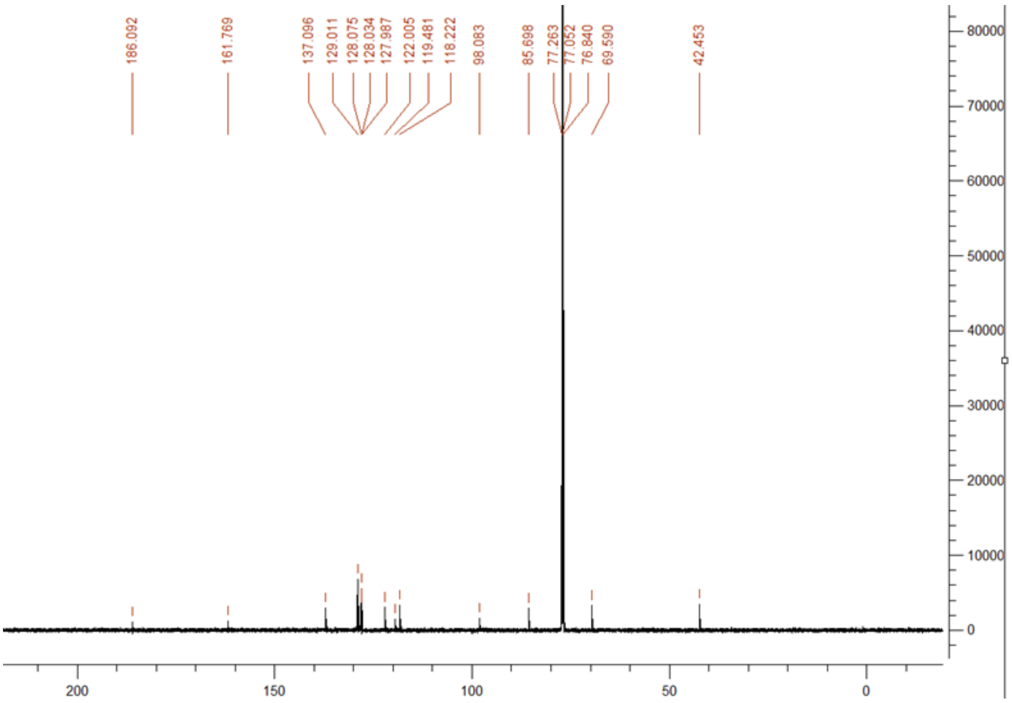

Figure S11a. ^{13}C **NMR spectrum of compound 8** ($CDCl_3$ 77.0 ppm)

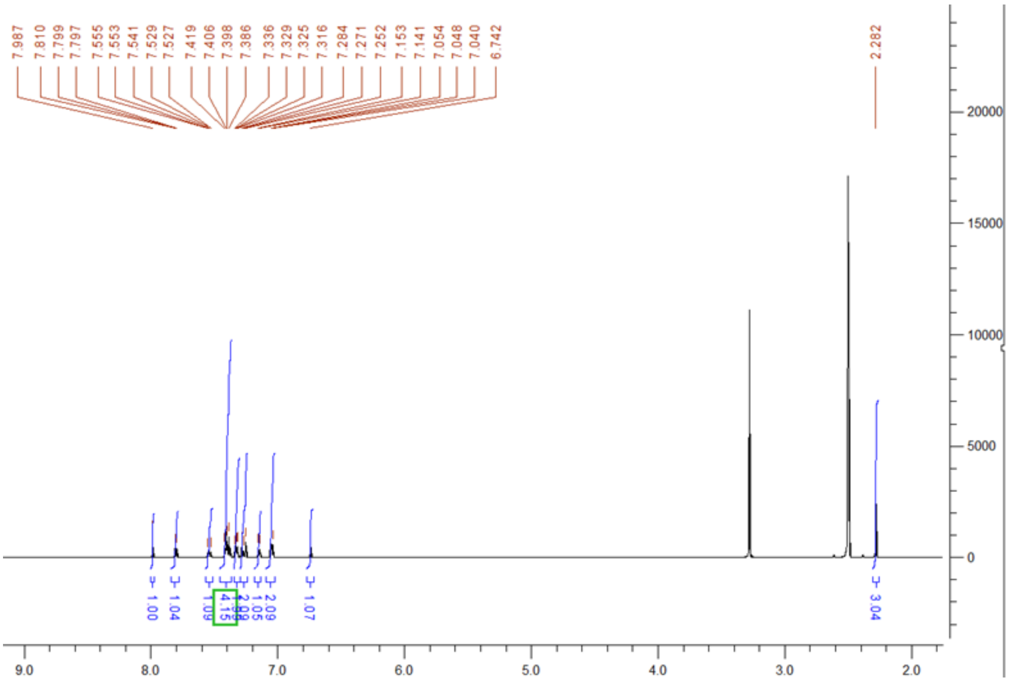

Figure S12. ^{1}***H*** **NMR spectrum of compound 9** (DMSO-d$_6$ 2.50 ppm; HDO 3.25 ppm)

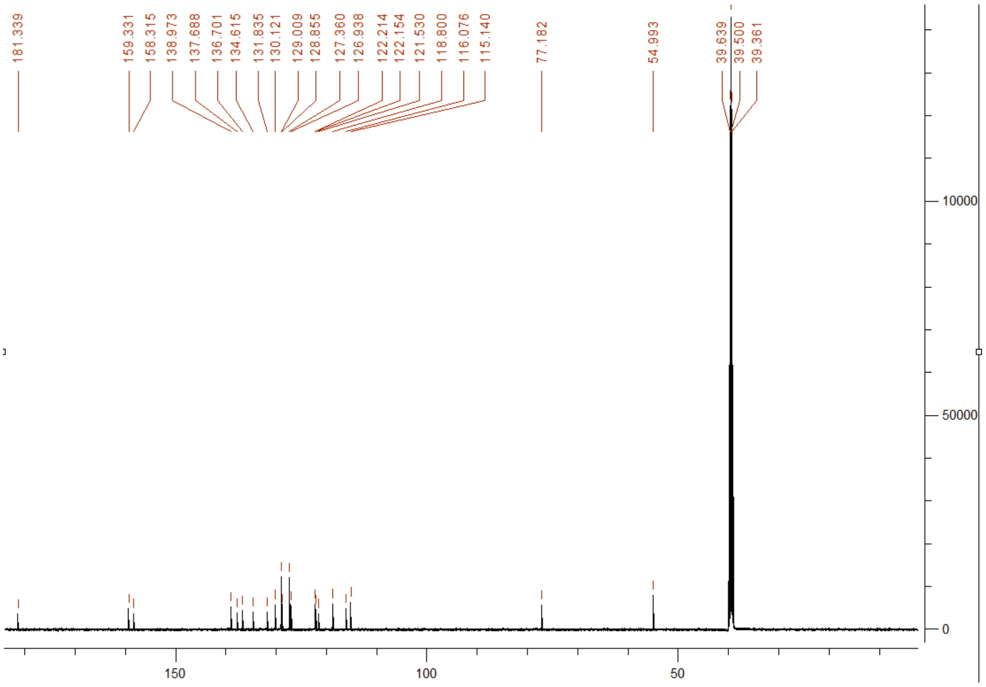

Figure S12a. ^{13}***C*** **NMR spectrum of compound 9** (DMSO-d$_6$ 39.5 ppm)

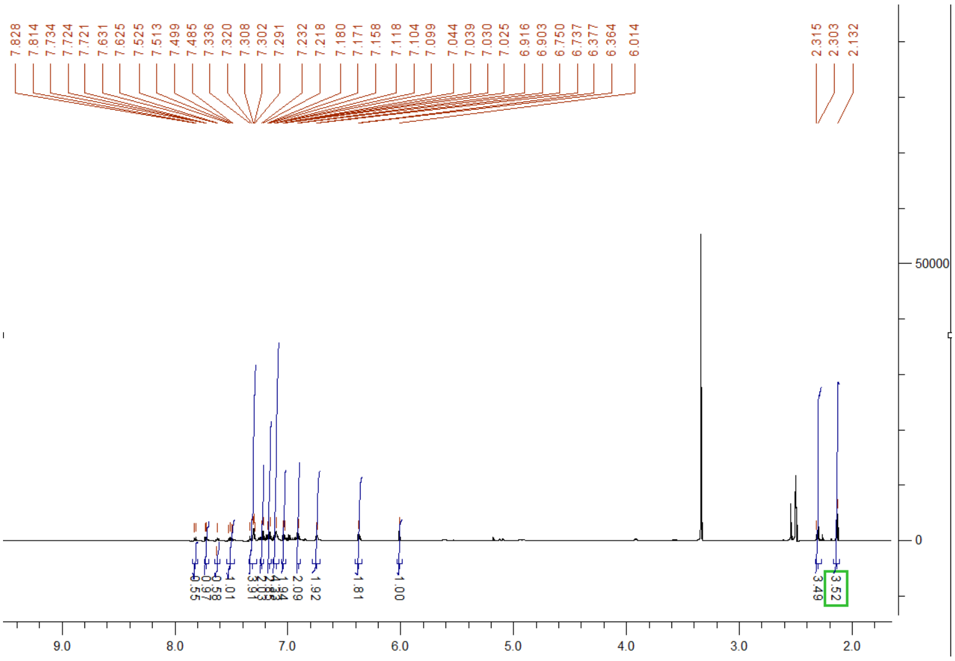

Figure S13. ^{1}*H* **NMR spectrum of compound 10** (DMSO-d$_6$ 2.50 ppm; HDO 3.25 ppm)

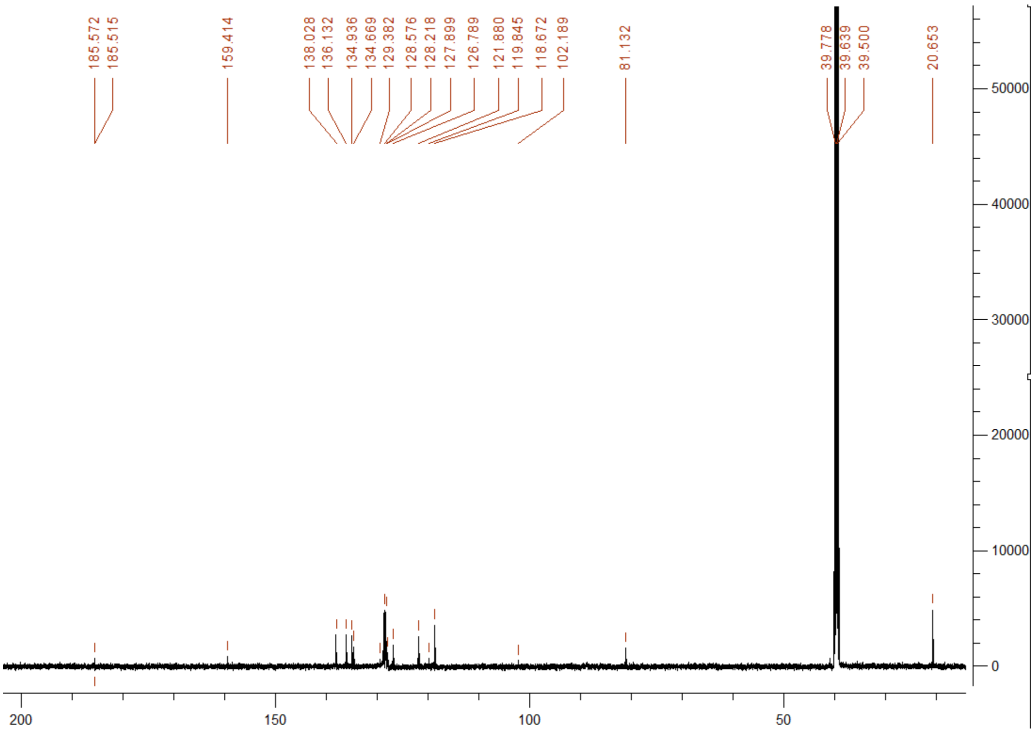

Figure S13a. ^{13}*C* **NMR spectrum of compound 10** (DMSO-d$_6$ 39.5 ppm)
